# EP27 Sjögren’s or not: an anxious diagnosis

**DOI:** 10.1093/rap/rkaa052.026

**Published:** 2020-11-03

**Authors:** Sharmin Nizam

**Affiliations:** Mid Yorkshire Trust, Wakefield, United Kingdom

## Abstract

**Case report - Introduction:**

Sjögren’s syndrome is a chronic, autoimmune condition usually characterised by reduced function of exocrine glands (mainly lacrimal and salivary) resulting in sicca symptoms. Affected patients may also have extra-glandular features including arthritis, neuropathy, and interstitial nephritis.

This is a case of possible Sjögren’s syndrome without classical features like positive serology or histology. This makes the patient feel anxious about his overall health. Diagnostic criteria have been debated over the years and whilst some clinical features may be suggestive, more objective evidence can help guide discussions on long term management and prognosis to allay anxiety.

**Case report - Case description:**

A 63-year-old Asian gentleman has had 6 years of intermittent cervical lymphadenopathy, dry eye and mouth symptoms without weight loss or respiratory complaints. His background includes ulcerative colitis (relatively stable), angina, hypertension, degenerative back pain (confirmed on MRI), dental extraction and diabetes.

Interval FNA sampling and excision biopsy of a prominent chain of right cervical nodes on separate occasions showed “reactive changes” with negative Mycobacterium TB screening (serology and lymph nodes).

Blood tests show a normal CRP (<5 mg/L), ESR 36 mm/h, raised polyclonal IgG 28.6 g/L (IgG subclass 1, 20.40 g/L, subclass 2, 9.36g/L, subclass 3, 0.954g/L, subclass 4, 9.430g/L) , normal complement and negative results for ANA, HLA B27, Anti CCP and ANCA.

Bilateral submandibular gland ultrasound showed hyperechoic lesions consistent with either chronic sialadenitis or Sjögren’s. FNA sampling of an intra-parotid lesion showed a “reactive” lymph node.

A left lower lobe 5mm calcified granuloma seen on plain film was confirmed on CT chest imaging along with mild inflammatory changes (lingual area) and multiple soft tissue density nodules up to 1cm in the anterior mediastinum. Initially thought thymoma related, later it was agreed these were benign lymph nodes after noting bilateral, sub-centimetre axillary and pre-tracheal nodes of similar appearance.

Following annual surveillance, a recent scan shows persistence of the lingular nodular focus, mediastinal lymphadenopathy and a 4mm ground glass nodule not thought suitable for PET CT or CT guided sampling. The previously seen parotid lymph node appears reduced and scattered low grade nodes are seen in the neck, chest, and porta hepatis.

Ophthalmologists note a poor-quality tear film with an equivocal Schirmer’s test. He has been treated for blepharitis and diagnosed with macular oedema. He was due to have a labial gland (lip) biopsy but later declined the procedure.

**Case report - Discussion:**

Sjögren’s syndrome has a female preponderance and is usually associated with sicca symptoms, a positive Schirmer’s test and autoantibodies (anti-Ro and anti-La). Extra-glandular features may exist, and secondary Sjögren’s features are seen in other autoimmune conditions.

Various diagnostic criteria have been proposed using clinical, serological, and/or histological features. This patient has sicca symptoms, lymphadenopathy, and imaging findings suggestive of Sjögren’s. Though not routinely used, salivary gland imaging features include enlarged, hyperechoic lesions and later stage multi-cystic or reticular patterns within atrophic glands.

Due to ethnicity, negative autoantibodies and imaging, the differential of tuberculosis (TB) was excluded. A labial gland biopsy was suggested as it may be a potentially sensitive and specific Sjögren’s biomarker. Presence of multiple, periductal, lymphocytic foci can help exclude alternative diagnoses like sarcoidosis, amyloidosis, or lymphoma. However, the patient declined the procedure due to concerns about possible post procedure hypersensitivity.

This patient has mild fatigue and non-specific arthralgia but not typical of fibromyalgia which is known to mimic Sjögren’s.

Reassuringly, he remains well but anxious about lymphadenopathy which he feels is unrelated to his mild ulcerative colitis managed with prednisolone enemas. In the absence of arthritis or significant organ involvement, he has only been given symptomatic treatment (e.g. eye drops). In Sjogren’s, any increased or persistent lymphadenopathy calls for further investigation. Other predictors include low complement and cryoglobulins which are absent in this patient.

This case may add to the evidence of co-existence of secondary Sjögren’s or Sjogren’s like syndrome with IBD which seems uncommon and in other cases, appears to be in conjunction with immunosuppressive treatment and autoantibodies.

Duration of follow up required remains uncertain and whilst the patient requires little ongoing monitoring, health anxieties can precipitate frequent contact.

**Case report - Key learning points**
 

- Sjögren’s syndrome (SS) can be variable in presentation but in most cases is mild
- Unlike other autoimmune disorders, in SS there is a lack of standardised criteria for diagnosis and classification
- Some features can be non- specific and like features of fibromyalgia and sarcoidosis
- In unclear cases, like this, objective markers like serology or histology (labial gland biopsy) may be more helpful
- In lymphadenopathy, depending on size and appearance, further investigations require multidisciplinary discussion to check if regular imaging is more appropriate compared to invasive tests. The frequency of imaging and potential radiation exposure needs careful consideration.
- In this case the patient is unwilling to undergo further invasive tests like a biopsy and the lymphadenopathy seen on imaging is thought relatively stable and not amendable to sampling.
- The ideal duration of follow up and need for ongoing investigations in this patient remains unclear – advice on monitoring and outcome of similar cases may help guide patient management and reduce anxiety

